# The ReFRAME library as a comprehensive drug repurposing library and its application to the treatment of cryptosporidiosis

**DOI:** 10.1073/pnas.1810137115

**Published:** 2018-10-03

**Authors:** Jeff Janes, Megan E. Young, Emily Chen, Nicole H. Rogers, Sebastian Burgstaller-Muehlbacher, Laura D. Hughes, Melissa S. Love, Mitchell V. Hull, Kelli L. Kuhen, Ashley K. Woods, Sean B. Joseph, H. Michael Petrassi, Case W. McNamara, Matthew S. Tremblay, Andrew I. Su, Peter G. Schultz, Arnab K. Chatterjee

**Affiliations:** ^a^California Institute for Biomedical Research, La Jolla, CA 92037;; ^b^Department of Integrative, Structural and Computational Biology, The Scripps Research Institute, La Jolla, CA 92037

**Keywords:** drug repositioning, phenotypic screening, *Cryptosporidium*, drug discovery, neglected tropical diseases

## Abstract

The chemical diversity and known safety profiles of drugs previously tested in humans make them a valuable set of compounds to explore potential therapeutic utility in indications outside those originally targeted, especially neglected tropical diseases. This practice of “drug repurposing” has become commonplace in academic and other nonprofit drug-discovery efforts, with the appeal that significantly less time and resources are required to advance a candidate into the clinic. Here, we report a comprehensive open-access, drug repositioning screening set of 12,000 compounds (termed ReFRAME; Repurposing, Focused Rescue, and Accelerated Medchem) that was assembled by combining three widely used commercial drug competitive intelligence databases (Clarivate Integrity, GVK Excelra GoStar, and Citeline Pharmaprojects), together with extensive patent mining of small molecules that have been dosed in humans. To date, 12,000 compounds (∼80% of compounds identified from data mining) have been purchased or synthesized and subsequently plated for screening. To exemplify its utility, this collection was screened against *Cryptosporidium* spp., a major cause of childhood diarrhea in the developing world, and two active compounds previously tested in humans for other therapeutic indications were identified. Both compounds, VB-201 and a structurally related analog of ASP-7962, were subsequently shown to be efficacious in animal models of *Cryptosporidium* infection at clinically relevant doses, based on available human doses. In addition, an open-access data portal (https://reframedb.org) has been developed to share ReFRAME screen hits to encourage additional follow-up and maximize the impact of the ReFRAME screening collection.

## Significance

The ReFRAME collection of 12,000 compounds is a best-in-class drug repurposing library containing nearly all small molecules that have reached clinical development or undergone significant preclinical profiling. The purpose of such a screening collection is to enable rapid testing of compounds with demonstrated safety profiles in new indications, such as neglected or rare diseases, where there is less commercial motivation for expensive research and development. Providing the academic and nonprofit research community access to a high-value compound collection and related screening data in an open-access platform should provide new tool compounds for biomedical research, as well as accelerate drug-discovery and/or development programs aimed at developing new therapies for diverse unmet medical needs.

In traditional drug-discovery workflow, hits identified by screening against large collections of small molecules require substantial preclinical optimization (e.g., potency, safety, pharmacokinetics), which significantly increases the resources, time, and risk associated with developing new medicines. Initiating drug-discovery campaigns from known drugs or advanced compounds with optimized pharmacokinetics and safety, rather than compounds with unoptimized properties and unknown liabilities that come from large-scale screens, can significantly reduce the resource burden and time associated with generating new clinical opportunities (1–3). Such an approach leverages prior investment in medicinal chemistry, pharmacology, and toxicology, which helps to focus, or even eliminate, resource-intensive chemistry and profiling assays that are common in small-molecule drug discovery (4). In addition, a newly discovered biological activity of a drug with a known mechanism of action can provide new insights into complex cellular biology, or even reveal previously unknown mechanisms by which a known drug can act. A number of impressive examples of repositioned drugs exist (e.g., thalidomide for multiple myeloma and sildenafil for erectile dysfunction), which reinforces the value of screening known drugs. Another key advantage is that the relatively small size inherent to repurposing libraries (e.g., thousands, rather than millions, of compounds) allows more complex biological assays with limited throughput to be deployed, for example, image-based screens involving whole organisms or primary cells. Indeed, such complex assays allow one to simultaneously interrogate large numbers of known and unknown targets that have likely never before been assayed against many known drugs.

While the rationale for this approach is widely acknowledged, an accessible, comprehensive set of such compounds is missing from the chemical libraries of most commercial and nonprofit drug-discovery organizations. While many academic and pharma groups have assembled sets of their own high–value-added compounds to accelerate internal drug-discovery efforts, these individual resources, in general, are not made available to the academic research community as tools to interrogate novel biology (1, 5). Even in instances where such compound sets are shared, legal agreements complicate collaborations and data sharing, and screening efforts are not typically coordinated such that comparisons across datasets are possible (6).

To address these critical gaps and enable drug repurposing efforts more broadly, particularly in disease areas with high unmet need and a paucity of new leads, an initiative called Repurposing, Focused Rescue, and Accelerated Medchem (ReFRAME) was undertaken. Herein, we describe a large-scale effort to generate a consolidated, high–value-added compound library as an open-access drug-discovery resource for academic laboratories around the world. Importantly, the purpose of this collection and the resulting screen data is to accelerate the development of new or existing therapeutics for unmet needs, and to enable public–private partnerships in situations where the drugs are still actively marketed. Drug-discovery efforts for neglected and rare diseases, which are often underrepresented in early stage screening campaigns, as well as phenotypic, image-based screens, such as screens using primary cells or disease models created from patient-derived induced pluripotent stem cells, could greatly benefit from such a widely available compound collection. The collection resides at a very manageable number of compounds (∼12,000), which is both conducive to the screening capabilities of many academic laboratories and amenable to medium-throughput assay formats (e.g., 96- or 384-well plate format) that do not require additional time-consuming assay optimization to miniaturize to a 1,536-well format. A screen of fewer than 30 plates in a 384-well format can provide promising tool compounds or leads poised for validation in animal efficacy studies. To exemplify this utility and applicability in neglected disease, we describe a cell-based screen of ReFRAME with an established assay that yielded screen hits supporting positive in vivo proof-of-concept experiments within 3 mo from screen initiation.

## Results

### Data Mining.

**Fig. 1.**
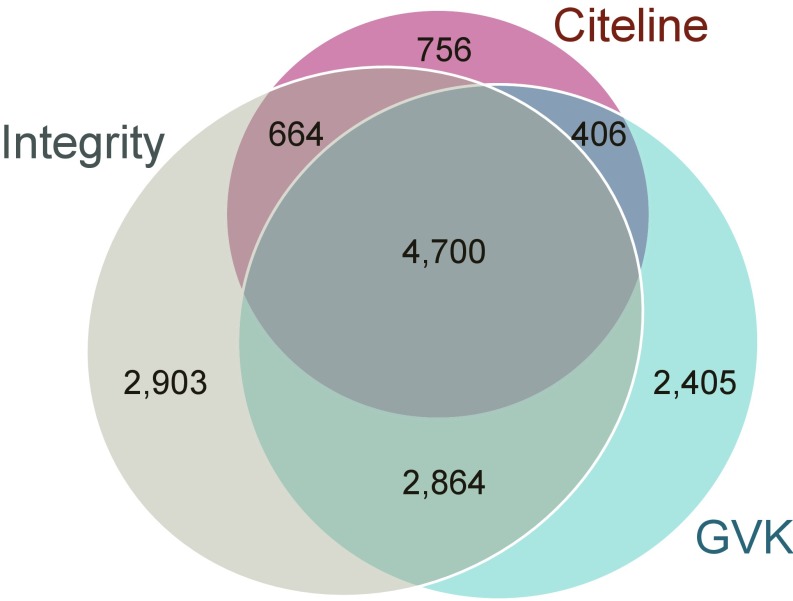
Overlap of drug entries across all three primary data sources.

The primary criterion applied for compound inclusion consideration in ReFRAME was whether clinical study data were available in at least one data source. As an exception, certain preclinical compounds under 1,500 Da with positive repeat in vivo animal efficacy and/or toxicity studies were also included. The first step in building this collection was to assemble a comprehensive cheminformatics library based on commercial drug databases. Data were initially pooled from three of the largest and most commonly used commercial databases, GVK Excelra GoStar (Global Online Structure Relationship Database), Clarivate Integrity, and Citeline Pharmaprojects, to arrive at a prioritized compound list of ∼14,000 compounds. These sources each has excellent stand-alone data curated from various sources including all available journals, patents, and other published literature. In addition, all three data sources have excellent web-based interfaces that allow for text-, structure-, and activity-based searching and the ability to export results. As an example of the breadth and depth of data in these sources, GVK GoStar has over 22 million structure–activity relationship (SAR) data points covering 6 million structures from 2.2 million patents and greater than 348,000 journal articles; it also includes rich lead optimization datasets with integrated clinical-stage compound data. The Excelra GoStar database includes a 5,000-approved compound drug database (encompassing 569 molecular targets) and a 29,000-compound clinical candidate compound collection. Clarivate Integrity and Citeline Pharmaprojects databases have comprehensive patent literature and chemical structure coverage, drawing upon broader sources such as patent applications, scientific conference presentations, and peer-reviewed literature. Citeline Pharmaprojects has over 35 y of drug development data from various sources including relevant pharmacokinetic and clinical trial information. Clarivate Integrity also has extensive drug information, with over 500,000 drug entries and over 74,000 assays containing more than 2.1 million SAR data points. Despite the size of these databases, Fig. 1 shows a surprising number of nonoverlapping entries across these data providers, further highlighting the need to interrogate multiple data sources. We therefore chose to combine data from these three sources to arrive at a consensus set of clinical and advanced preclinical compounds.

### Data Curation.

For each data provider, the list of clinical compounds with known structures was filtered to remove drug combinations of individual compounds already included in the collection, isotopically labeled compounds, diagnostic agents, and chemosensitizers (which collectively totaled 1,299 entries). The filtered lists were then combined, salt forms were separated from active drug SMILES (simplified molecular-input line-entry system) (for 3,344 entries), and duplicate entries were deleted to generate an initial list of 13,904 compounds targeted for inclusion in ReFRAME. An internal identifier was next assigned to each structure that was listed as being a clinical-stage compound. Subsequently, a table was generated, tying each of the internal identifiers to the respective data provider records. The ReFRAME compound list took the data provider compounds by desalting the SMILES strings, converting to International Chemical Identifier (InChI) keys, and merging based on exact matches to the InChI keys. Additional internal identifiers were subsequently added for preclinical compounds targeted for inclusion. The final list also includes prodrugs and active metabolites of clinical compounds listed as preclinical by the data providers and compounds recently advanced to the clinic but not yet listed as clinical in the databases.

Stereochemical configuration required careful curation to accurately reflect the active stereoisomers of compounds chosen for inclusion in ReFRAME. For 2,816 data entries, stereochemistry was either underspecified (e.g., no absolute configuration was indicated for chiral sp3 centers) or overspecified (e.g., double-bond stereochemistry was indicated in cases where the experimental compound is in fact a mixture). For 750 underspecified compounds that were not commercially available for purchase, the stereochemistry of the experimental compound was manually researched and confirmed before synthesis. Where a racemic mixture was synthesized and separated by chiral purification to obtain the desired experimental compound, its corresponding stereoisomer(s) was typically included in the collection as well to better understand the role of stereochemistry for hits in the repurposing assay.

### Determining “Best Guesses” for Unannotated Compounds.

In some cases, actual structures of early clinical-stage compounds are not explicitly disclosed in the literature (2,204 compounds). However, collective examination of the patent literature (based on claim structure and subsequent process patents) and public data disclosures can allow one an educated guess of the structure; inclusion of these compounds adds significant value to this compound collection. While there is a risk that the compound selected will not be the actual structure of a drug, a close chemical analog can still serve as a valuable starting point for hit follow-up. This process was completed in two rounds of initial searching followed by more in-depth analysis and provided 794 additional chemical structures. Reference materials were cited in the “best-guess” section of the data portal as outlined below.

### Compound Acquisition and Screening Platform Format.

From the list of ∼14,000 entries with identified structures, structure- and name-based searches were used to source commercially available compounds, resulting in 6,805 compounds purchased from vendors at ∼20-mg scale. This amount of material is enough for over 6,000 screens in a 384-well plate format using a typical screening volume (20 to 50 μL). Those compounds that were not commercially available were synthesized at multiple contract research organizations (CROs) in parallel; the data portal includes annotation to facilitate correspondence with the CROs for synthetic details or resupply. In total, 4,194 compounds with known structures were successfully synthesized and are currently included in ReFRAME. Of the 794 compounds with best-guess structures identified, 522 have to date been synthesized and included in the screening collection. Finally, we acquired 427 close analogs that were synthesized from common intermediates in the course of synthesizing the parent drug. These close analogs, as well as related stereo- and regioisomers, were opportunistically included, providing some level of SAR directly from the screen, enriching and expediting follow-up activities. Each CRO has agreed to provide information—including cost for resupply, analytical data, and synthetic procedures—on an as-needed basis. A summary of compound procurement and ReFRAME composition is shown in Table 1.

**Table 1.** Compositional breakdown of the ReFRAME compound library

Description	No. of entries
Total entries with structure in three drug databases	13,904
Purchased compounds from commercial sources	6,805
Synthesized compounds from disclosed structures	4,194
Entries without structure annotation	2,204
Structure-elucidated	794
Elucidated structures synthesized	522
Close analogs in collection as part of synthesis efforts	427
Total ReFRAME compounds plated and ready to screen to date	11,948

Once compounds were procured, quality control (QC) was performed by liquid chromatography-mass spectrometry (LC-MS) and/or NMR when required. Compounds exhibiting less than 95% purity by LC-MS were purified further to achieve a minimum value of 95% purity determined by LC-MS and ^1^H-NMR. Approximately 20% of all synthesized compounds were analyzed by ^1^H-NMR to confirm that the material matched literature specifications. After QC was completed, compounds were plated in batches at 2 mM concentration in DMSO for a 1,536-well screen format and 10 mM concentration for a 384-well screen format using a robotic system (based on a HighRes Biosolutions Dual NanoCell) and accessory software that integrates acoustic compound dispensing (Labcyte Echo 555). Auxiliary instruments for compound management (liquid handlers, integrated analytical balance, and volume-check device) were tuned to enable us to provide single-use copies (typically 10- to 200-nL aliquots) of the collection for external distribution in either 384- or 1,536-well plates for primary screens. Compounds not soluble in DMSO were plated in water (129 compounds); compounds lacking long-term solubility in DMSO were suspended just before dispensing to avoid precipitation (71 compounds).

**Fig. 2.**
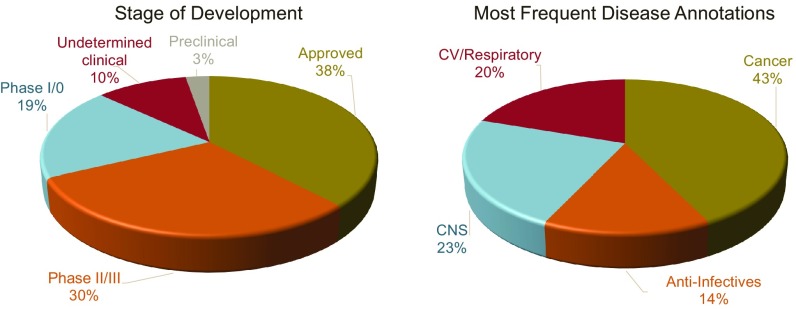
Distribution of ReFRAME entries across stages of clinical development and disease indications. CV, cardiovascular.

To date, ∼12,000 compounds have been sourced, received, and plated for screening. Compounds spanning all phases of clinical development are represented in the collection (Fig. 2). Over 59% of the collection is made up of experimental drugs [i.e., not Food and Drug Administration (FDA)-approved] with significant human safety data, offering the opportunity to delve deeper than conventional FDA-approved libraries to repurpose and quickly progress compounds in a new indication or to discover new therapeutically relevant biology. In addition, the compound collection has significant representation from all disease areas with a historical commercial focus including oncology, immunology, infectious disease, and cardiovascular disease. This compound collection encompasses other previously described collections based on similar clinical data sources (1) but also includes nearly 5,000 synthesized compounds not in any other public collections. Also, 12% of the chemical structures in this collection are not contained in PubChem.

### ReFRAME Screen Identifies Novel Candidate Therapeutics for *Cryptosporidium* Infection.

To exemplify the utility of this 12,000-compound set, we screened it for previously unknown inhibitors of *Cryptosporidium* proliferation (i.e., cryptosporidiosis). *Cryptosporidium* spp. are an apicomplexan protozoan that is an important cause of diarrhea in humans and some domestic animals. The parasite relies on an oral–fecal route of transmission, typically through ingestion of contaminated water or food. Ingested oocysts are activated to release sporozoites, which invade host epithelial cells in the small intestine, where they undergo replication and sexual differentiation back into oocysts to complete the life cycle. A large molecular epidemiology study, the Global Enteric Multicenter Study, revealed *Cryptosporidium* spp. to be the second-leading cause of life-threatening diarrheal disease in young children (7). Currently, there is only one approved drug to treat *Cryptosporidium* infection—nitazoxanide, which has modest potency and is ineffective in patients with compromised immune systems. Because there is little commercial interest in this pathogen, limited drug discovery has been conducted and few if any validated drug targets exist (8, 9). Therefore, the identification of safe and effective clinically approved molecules for the treatment of infection in young children and immunocompromised populations would be highly advantageous.

**Fig. 3.**
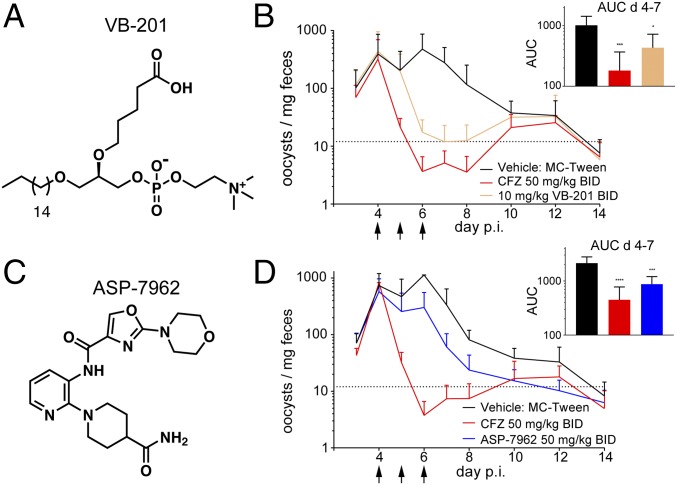
ReFRAME library compounds with anticryptosporidial efficacy. (*A* and *C*) The structures of oxidized phospholipid VB-201 and TrkA inhibitor ASP-7962. ASP-7962 is a best-guess structure from a phase 2 trial. (*B* and *D*) Oocyst shedding trace of mice infected with 1 × 10^6^ oocysts on day 0, then monitored for 2 wk. Line graph data are weight-adjusted mean oocyst counts ± SD (*n* = 8 over two separate experiments). (*B* and *D*, *Insets*) Bar graphs are oocyst counts ± SEM (*n* = 4) measured on day 7. These data were assessed with one-way ANOVA. Treatments were compared with the previously investigated compound clofazimine (CFZ) (50 mg/kg BID). AUC, area under the curve. *P < 0.05; ***P < 0.005; ****P < 0.001.

A high-content screen of *Cryptosporidium parvum*-infected human intestinal epithelial adenocarcinoma (HCT-8) cells, previously developed to identify *C. parvum* proliferation inhibitors, was used to screen the ReFRAME library (10). Of the 73 selective, submicromolar hits resulting from the screen, two novel compounds with promising profiles were identified, as well as the rediscovery of clofazimine, an FDA-approved antibiotic for the treatment of leprosy, which we identified previously as a repositioning candidate for cryptosporidiosis (10). VB-201 (EC_50_ 860 nM; structure shown in Fig. 3*A*) and ASP-7962 (EC_50_ 54 nM; structure shown in Fig. 3*C*) are clinical-stage compounds with promising activities. VB-201 is a choline-based phospholipid that binds directly to Toll-like receptor-2 (TLR-2) and CD14, a coreceptor of TLR-4, inhibiting downstream signaling and cytokine production. This compound is currently under investigation as a potential therapeutic for nonalcoholic steatohepatitis and liver fibrosis (11), atherosclerosis (12), and several other immune-related disorders at doses of up to 160 mg/d (13). ASP-7962 is a best-guess structure of a TrkA receptor antagonist that has advanced into a phase 2 clinical trial to assess its analgesic properties in patients with pain from osteoarthritis of the knee at 50 mg/d (14). The mechanisms by which these compounds exhibit anticryptosporidial activity is unclear at present. While they have annotated activity against human targets and may modulate host functions that subsequently affect intracellular infection of the parasite, it is also possible that these compounds have direct effects on the parasite, presumptively “off-target” relative to their clinical indications. This is especially likely for VB-201, which targets an immune signaling pathway that is not recapitulated in the simple cell-based screen assay composed of the human intestinal adenocarcinoma cell line HCT-8.

The activity of these compounds was confirmed in an in vivo disease model. The juvenile IFN-γ^−/−^ mouse is susceptible to oral *C. parvum* infection (15), and has been previously used as an acute model of cryptosporidiosis, characterized by a period of intense oocyst fecal shedding 3 to 8 d post infection (p.i.). This reproducible fecal oocyst shedding is measured by flow cytometry to quantify therapeutic efficacy of experimental compounds from the start of treatment (day 4 p.i.) until self-resolution (days 10 to 12 p.i.). Nitazoxanide, the standard of care, does not show efficacy in murine models of cryptosporidiosis, and therefore compounds of interest are benchmarked to clofazimine in this model (10). Six total oral doses of 10 mg/kg VB-201 or 50 mg/kg ASP-7962 were given twice per d (BID) for 3 d beginning at the onset of high-level oocyst shedding (day 4 p.i.). Controls included a group of vehicle-treated animals and a group of clofazimine-treated animals (50 mg/kg BID). Drugs were administered as methylcellulose (MC)/Tween suspensions (or blank MC/Tween for vehicle controls) at allometrically scaled doses based on extrapolation of available clinical dose levels (human and rodent pharmacokinetic data are not publicly available). Throughout the active shedding period (days 4 to 8 p.i.), and before self-resolution, VB-201 reduced oocyst shedding by >2 log_10_ (100-fold) at day 7 and subsequently to below the reliable limit of detection by the final day of treatment (Fig. 3*B*). A snapshot analysis of oocyst shedding on day 7 p.i. shows a statistically significant decrease in shedding (*P* = 0.028; Fig. 3*B*, *Inset*) from the vehicle-treated, infected control group. Similarly, in a separate study, ASP-7962 treatment resulted in an approximate 1-log_10_ (10-fold) reduction in oocyst shedding at day 7 compared with the vehicle-treated control group, showing a statistically significant decrease in oocyst shedding (*P* = 0.0004; Fig. 3*D*, *Inset*). The efficacy of both of these compounds in this model is better than nitazoxanide.

### Development of a Public Data Repository.

To facilitate dissemination of ReFRAME screen results to the broader research community, we created an online portal, available at https://reframedb.org. This portal serves two main functions. First, the portal provides continuously updated information on the ReFRAME compound library. The identity of all clinical compounds from data vendors, including removal of non–small-molecule drug data curation work described previously, is provided through the portal. Additional compound annotation relating to, for example, mechanism of action, known targets, drug categories, route of administration, and phase of approval, is also included. Second, the portal will provide access to screening results, including those described in this paper. Users can search the collection by compound, browse by activity (e.g., in the *Cryptosporidium* screen), or download these data for offline analysis. In addition to disseminating biomedical data, the portal also serves as a mechanism to solicit compound annotation from the community. This portal is based in part on Wikidata, a centralized, open, and community-maintained knowledge base (16).

In addition to the *Cryptosporidium* screen, the portal also displays screen hit results from several additional screens relating to diseases of the developing world (e.g., mycobacteria, poliovirus, and malaria parasites). As shown in Table 2, active ReFRAME hits were found for multiple pathogens and are currently being characterized. We intend to conduct many more screens and envision sharing ReFRAME with collaborators around the world via a simple material transfer agreement, which contains a commitment to global access and provides for inclusion of data in the portal on a time frame intended to be meaningful for the scientific community.

**Table 2.** Brief overview of cell-based and biochemical assays against diverse pathogens

Assay	Confirmed hits
*Cryptosporidium*: 1536w *C. parvum* high-content imaging proliferation assay	119
*Giardia*: 1536w luciferase assay	57
Helminth: 1536w Wolbachia elimination assay in *Drosophila* cells	175
Malaria: 1536w delayed death inhibitor assay	89
Mtb: intramacrophage inhibitor	84
*Poliovirus*: 1536w 48-h cytopathic effect assay (HeLa S3, Hep2, Vero)	82
*P. falciparum* TyrRS (biochemical)	41
Mtb OxPhos (biochemical)	50
Mtb Rho (biochemical)	85
Mtb: glucose-supplemented broth	224
Zika virus inhibitor [Vero/(SH-SY5Y)]	18

## Discussion

We have generated a library of ∼12,000 high-value compounds composed of purchased or resynthesized FDA-approved drugs (38%), as well as investigational new drugs currently or previously in any phase of clinical development (59%), including 522 noncommercially available compounds that represent best guesses of undisclosed structures. ReFRAME is unique among repurposing collections because of its scale and comprehensiveness, representing a singular resource for the biomedical research community. ReFRAME was generated by systematically querying available clinical data to obtain compounds without regard to mechanism of action, availability, patent status, or cost. This effort involved first determining a consensus set of registered and investigational drugs, which required collecting data from multiple data sources including GVK Excelra GoStar, Clarivate Integrity, and Citeline Pharmaprojects, followed by significant manual curation to identify structures of compounds that are not sufficiently specified or disclosed in the literature. Comparison with a large third-party drug database revealed no new entries relative to our final compound list, suggesting that our data-mining approach was comprehensive. Approximately half of the ReFRAME collection was acquired through purchase from commercial vendors. The remainder of the collection was generated by a major concerted effort of half a dozen CROs over a period of 24 mo, resulting in the successful delivery of over 5,197 compounds in >20-mg quantities derived from multistep syntheses as complex as 30 steps.

A screening collection of 12,000 small-molecule compounds is a manageable set of molecules for most academic laboratories to screen in cell-based assays in a 384- or 1,536-well plate format. We assembled the screening library in sufficient quantities to support a large number (>6,000) of primary screens, as well as secondary functional assays, to validate hits from novel cell-based screens. For studies that require larger quantities of compounds (e.g., in vivo studies), investigators can directly engage vendors for resupply, taking advantage of the investment made in optimizing synthetic protocols during the production of ReFRAME. Importantly, this library follows the global access policies set forth by the Bill & Melinda Gates Foundation, and all screen hit information will be provided in a timely manner to an open-access database (https://reframedb.org) to promote data sharing and drug-discovery opportunities.

One key element in successful drug repurposing is the ability to translate

in vitro data into in vivo proof-of-concept efficacy in relevant animal models. In many cases, one can use a strict downselection strategy from doses safely achievable in humans to allometrically determine the relevant drug levels to target in rodent efficacy studies. In some cases, we find that safe, desirable plasma levels that match the in vitro potency cannot be realized by direct compound repurposing, requiring a focused medicinal chemistry effort to synthesize derivatives to improve potency or to remove liabilities of the known drug (17).

To illustrate the utility of ReFRAME, we identified two screen hits demonstrating efficacy in a rodent model of cryptosporidiosis. Both VB-201 and a presumed structure for ASP-7962 were found to be potent inhibitors of parasite growth in vitro at concentrations that could be safely achieved in the mouse model. For comparison, KDU731 and BKI 1369 are two of the most advanced anticryptosporidial agents being developed. KDU731 was derived from a series of inhibitors of *Plasmodium* spp. phosphatidylinositol-4-OH kinase (18), and BKI 1369 putatively targets calcium-dependent protein kinase 1, based on its activity against other apicomplexan parasites (19, 20). Unlike the compounds we describe herein, which can be directly evaluated in patients, both KDU731 and BKI 1369 required intensive medicinal chemistry and still require time- and resource-intensive animal safety and toxicity profiling before entering the clinic as a new chemical entity for the treatment of cryptosporidiosis. Additional screens have been completed on a variety of pathogens providing >1,000 reconfirmed hits over 11 screens that are currently being triaged for in vivo confirmation. Our expectation is that additional screening of this modestly sized compound set will yield additional clinically relevant repurposing opportunities.

Limitations on the logistical supply of the library and preexisting intellectual property of compounds included in the ReFRAME collection are important screening considerations. In anticipation of conducting hundreds of screens, the strong preference is to provide assay-ready plates to external screening partners to minimize compound waste. Ideally, an entire screening effort (defined as a primary screen in single replicate and an eight-point dose–response reconfirmation in duplicate) would consume a maximum of 600 nL 10 mM library stock per screen hit. This approach to conserving compound quantities to maximize screening longevity precludes the distribution of library copies to interested screening parties. As a consequence, the compound quantity restrictions may impose limitations on the timing of compound addition to the assay and screens with long-duration assays that require media changes. The second major consideration is whether screen hits may be limited by existing intellectual property. The inclusion of actively developed clinical candidates and recently approved drugs may limit the freedom to operate on compound repurposing. However, each patent-limited discovery will need to be evaluated on a case-by-case basis and, where appropriate, the library provides an opportunity to engage the patent holders for discussion. Since neglected tropical diseases are a primary focus of this library, we foresee less interference for noncommercial drug repurposing in resource-limited countries.

Efforts are ongoing to synthesize an additional ∼2,000 entries targeted for inclusion as a means of further augmenting the collection and adding compounds that have been advanced into clinical development since the inception of the ReFRAME initiative where the exact chemical structures are not readily ascertained. Another ongoing need will be to generate more copies of the libraries to broadly distribute within the academic community and other nonprofit drug-discovery organizations. Our hope is that by making ReFRAME available as a public resource, we will accelerate the development of new therapies to treat unmet medical needs and provide valuable tool compounds with in vivo activities to reveal new insights into disease biology. The drug repurposing portal will document ReFRAME’s impact by providing a continuously growing set of highly curated screening data to the scientific community in the area of neglected tropical diseases.

## Materials and Methods

### Drug Database Informatics.

Comparisons of chemical structures to find exact matches and similar matches in high throughput were conducted using Open Babel software (21, 22), version 2.3.2, as modified by the authors for correctness (21, 23) and performance (24, 25). For similarity searches, the Tanimoto coefficient on FP2 fingerprints was used. Tautomers were detected by using Open Babel’s obtautomer to enumerate all tautomers and taking the first tautomer in ASCII string order as the canonical tautomer. Tautomers not recognized by this method were further recognized by manual inspection based on similarity of either structure or synonyms or, as a last resort, by our chemical registration system. Manual inspection of large compounds to identify differences was facilitated by ChemDraw 13.0.2 (PerkinElmer) and AccelrysDraw 4.2 (BIOVIA). Analysis of string similarity between lists of synonyms was conducted using the pg_trgm extension (26) of the PostgreSQL 9.4 Database System, as modified by the authors for performance (27), later changing to PostgreSQL 9.6.

### *Cryptosporidium* in Vitro Assay.

A 1,536-well cell assay was used to screen the ReFRAME library against *C. parvum* as previously described (10). Briefly, human ileocecal adenocarcinoma (HCT-8; ATCC CCL244) cells were plated (5 µL per well) into 1,536-well assay plates at a density of 5.5 × 10^5^ cells per mL (2,750 cells per well). Cells were allowed to grow for 24 h at 37 °C with 5% CO_2_ in a humidified tissue culture incubator. *C. parvum* oocysts (Iowa strain; Bunch Grass Farm) were excysted and prepared for inoculation as previously described (28). The oocysts were diluted with assay medium to 1.04 × 10^6^ oocysts per mL (3,125 oocysts per well) and dispensed (3 and 8 µL per well final volumes) with a MultiFlo FX Multi-Mode Dispenser (BioTek). Plates were then spun at 150 × *g* for 3 min in a Sorvall Legend XTR benchtop centrifuge (Thermo). Infected cells were incubated at 37 °C with 5% CO_2_ in a humidified tissue culture incubator covered with metal assay lids (The Genomics Institute of the Novartis Research Foundation) for 48 h. Following incubation, infected cells were fixed and stained, and then imaged on a CellInsight CX5 High Content Screening Platform (Thermo) with a 10× objective as previously described. The plates were imaged with a CellInsight CX5 High Content Screening Platform (Thermo) with a 10× objective. Both cytotoxicity against HCT-8 cells (number of nuclei relative to DMSO-treated controls) and *Cryptosporidium* inhibition (spot counts relative to DMSO-treated controls) were assessed.

Images were processed by HCS Studio Scan software, and selected object count (HCT-8 cells) and spot count (*Cryptosporidium*) were analyzed in Genedata Screener (v13.0-Standard). Spot count and selected object count were normalized to neutral controls minus inhibitors (floxuridine for spot count, and puromycin for selected object count). Dose–response curves were fit with Genedata Analyzer using the Smart Fit function. Final filtered hits included those with an EC_50_ (half-maximal effective concentration) ≤1 µM, with a CC_50_ (half-maximal cytotoxic concentration) ≥10-fold greater than the EC_50_ value.

### Mouse Cryptosporidiosis Model.

Four-week-old female C57BL/6 IFN-γ^−/−^ mice were purchased from the Jackson Laboratory and infected with *C. parvum* oocysts (Iowa strain; Sterling Parasitology Laboratory, University of Arizona) as previously described (10). Briefly, *C. parvum* oocysts were adjusted to a final density of 5 × 10^6^ per mL in cold, sterile, distilled water. Mice were infected via oral gavage with 200 μL (10^6^ oocysts) using a 20-gauge by 1.5″ feeding needle. Twice per d on days 4, 5, and 6 post infection, mice were gavaged with 10 mL/kg of either vehicle (0.5% methylcellulose/0.5% Tween 80 in water), 50 mg/kg clofazimine (positive control), 50 mg/kg ASP-7962, or 10 mg/kg VB-201 (*n* = 4 per group). Data shown are two independent experiments of the same dosing regimens combined (*n* = 8 total). Each day, between days 3 and 14 post infection, mice were temporarily placed in isolation to allow for collection of three fecal pellets per mouse. Pellets were weighed and placed in 0.5 mL 2.5% potassium dichromate solution, and stored at 4 °C until processed. For quantification of oocyst shedding in feces, oocysts were isolated using a modified discontinuous sucrose gradient technique (29). The extracted oocysts were then incubated for 30 min at room temperature with 0.25 μg fluorescein isothiocyanate-conjugated mouse anti-*Cryptosporidium* antibody (OW50-FITC; Bio-Rad, 2402-3007), and diluted to 200 μL with PBS. Samples were analyzed using a Guava EasyCyte flow cytometer and CytoSoft Data Acquisition and Analysis software (v5.3; Guava Technologies), using a 100-s sampling interval, 0.59 μL/s flow rate, and logical gating of forward and side light scatter and OW50-FITC fluorescence signals. Final graphing and statistical analyses of these data were done using Prism software (v6; GraphPad Software).

### Data and Materials Availability.

Materials must be obtained through a Materials Transfer Agreement (MTA), and drug information and screening data are available in the ReFRAME portal (https://reframedb.org).
